# Rationale and design of a complex intervention measuring the impact and processes of social accountability applied to contraceptive programming: CaPSAI Project

**DOI:** 10.12688/gatesopenres.13075.2

**Published:** 2020-12-10

**Authors:** Petrus S Steyn, Victoria Boydell, Joanna Paula Cordero, Heather McMullen, Ndema Habib, Thi My Huong Nguyen, Dela Nai, Donat Shamba, James Kiarie, CaPSAI Project

**Affiliations:** 1 UNDP/UNFPA/UNICEF/WHO/World Bank Special Programme of Research, Development and Research Training Human Reproduction, Avenue Appia 20, Geneva, 1202, Switzerland; 2 Global Health Centre, Geneva Graduate Institute, Maison de la Paix, Chemin Eugène-Rigot 2A, Case Postale 1672, Geneva, 1211, Switzerland; 3 Centre for Global Public Health, Institute of Population Health Sciences, Queen Mary University of London, 58 Turner Street, London, E1 2AB, UK; 4 Population Council, 204 Yiyiwa Drive, Abelemkpe, Accra, Ghana; 5 Department of Health Systems, Impact Evaluation and Policy, Ifakara Health Institute, P.O.BOX 78373, Dar es Salaam, Tanzania

**Keywords:** Protocol, social accountability, community monitoring, contraception, complex intervention

## Abstract

**Background**: There are numerous barriers leading to a high unmet need for family planning and contraceptives (FP/C).  These include limited knowledge and information, poor access to quality services, structural inefficiencies in service provision and inadequately trained and supervised health professionals. Recently, social accountability programs have shown promising results in addressing barriers to accessing sexual and reproductive health services. As a highly complex participatory process with multiple and interrelated components, steps and actors, studying social accountability poses methodological challenges. The Community and Provider driven Social Accountability Intervention (CaPSAI) Project study protocol was developed to measure the impact of a social accountability intervention on contraceptive uptake and use and to understand the mechanisms and contextual factors that influence and generate these effects (with emphasis on health services actors and community members).

**Methods**: CaPSAI Project is implementing a social accountability intervention where service users and providers assess the quality of local FP/C services and jointly identify ways to improve the delivery and quality of such services. In the project, a quasi-experimental study utilizing an interrupted time series design with a control group is conducted in eight intervention and eight control facilities in each study country, which are Ghana and Tanzania. A cross-sectional survey of service users and health care providers is used to measure social accountability outcomes, and a cohort of women who are new users of FP/C is followed up after the completion of the intervention to measure contraceptive use and continuation. The process evaluation utilizes a range of methods and data sources to enable a fuller description of how the findings were produced.

**Conclusion**: This complex study design could provide researchers and implementers with the means to better measure and understand the mechanisms and contextual factors that influence social accountability processes in reproductive health, adding important findings to the evidence base.

## Abbreviations

CaPSAI, Community and Provider driven Social Accountability Intervention; CRF, case report form; CSC, community score cards; CSO, civil society organization; EDC, electronic data capture; FP/C – family planning/contraception; GEE, generalized estimating equation; GII, Ghana Integrity Initiative; IDI, in-depth interviews; IHI, Ifakara Health Institute; ITS-CG, interrupted time series with a control group; MRC, Medical Research Council; NMRI, National Medical Research Institute; NON, non-participant observation; SRH/HRP, Department of Sexual and Reproductive Health, which includes the United Nation Development Program/United Nations Population Fund/United Nations Children’s Fund/WHO/World Bank Special Programme of Research, Development and Research Training Human Reproduction; SRH/SIS, Department of Sexual and Reproductive Health’s Statistics and Informatics Service; RMNCAH, reproductive, maternal, newborn, child and adolescent health; RCT, randomized control trial; ToC, theory of change; WHO, World Health Organization.

## Introduction

Many women who want to use family planning and contraceptives (FP/C) are unable to access it and are at risk of unintended pregnancy ^1^. Limited knowledge and information about FP/C, poor access to quality services, poor quality of care and client-provider interaction, structural inefficiencies in service provision and untrained health professionals remain barriers for those seeking services ^2^. Interventions that change the attitude, norms, and behavior of both service users and providers, such as health communication, community group engagement, addressing provider bias and improving client-provider interactions, have proven effective in addressing these barriers ^3, 4^.

These are interventions encompassed within social accountability processes, where a combination of activities aims to empower and educate clients to demand quality services and to support health services actors to recognize and act on citizens’ demands ^5^. These activities include civic and health education, group priority-setting, joint problem identification, and problem-solving. At the heart of these actions are efforts to change: 1) values, norms, and culture related to health-seeking; 2) attitudes and perceptions; and 3) resources and capacities of the health service. These actions change the behavior of service users and providers and, in turn, contribute to improvements in service availability, access, utilization and quality of services ^6^. 

Not only do these processes have instrumental value but they also have intrinsic importance as well. Participation is central to a rights-based approach and to the provision of contraceptive services ^7– 10^. In recent years, a number of social accountability programs have shown promising results in the field of sexual and reproductive health ^3, 11, 12^. Social accountability programs evaluated have been effective in: increasing service utilization; better service delivery; improved health provider responsiveness; increasing knowledge and information; governance; and in health outcomes in broader reproductive, maternal, newborn, child and adolescent health (RMNCAH) ( Table 1).

**Table 1.** Examples of reported outcomes from studies of social accountability in reproductive, maternal, newborn, child and adolescent health (RMNCAH).

Outcome area	Reported specific outcomes
Service utilization	Increased immunizations ^14^ Increased examinations ^14^ Improved access to services ^15^ Increased antenatal visits ^16^ Use of skilled birth attendants ^16^
Service delivery	Available medical equipment ^16, 17^ Available supplies ^14, 16, 18^ Increased resources ^19^ Increased staffing ^17^ Improved infrastructure ^17^ Reduction in waiting time ^18^
Service providers	Less absenteeism ^16, 18^ Improved morale ^19^ Improved training and supervision ^19^ More engaged provider-client interaction ^17^
Knowledge and information	Safe sex/high risk behavior ^20, 21^
Governance	Participation ^17^ Transparency ^17^ Community and decision- making engagement ^17^
Health outcomes	Child weight ^16^ Maternal mortality ratio ^22^

### Rationale

Although there are some data supporting the implementation of social accountability programs for FP/C ^3, 11, 13^, there remains little robust evidence documenting and demonstrating the effects of these changes as applied to FP/C service delivery.

Social accountability is a highly complex participatory process with multiple and interrelated components, steps and actors and several simultaneous processes of behavior change that create methodological challenges for research ^23– 25^. The complexity of the interventions and the range of interrelated outcomes of interest create challenges for conceptualizing research and developing appropriate study designs ^23, 26^.

The Community and Provider driven Social Accountability (CaPSAI) Project builds on and contributes to a growing but limited body of work that aims to better understand how social accountability and participatory processes in the context of an FP/C program contribute to people enjoying the highest possible sexual and reproductive health and contribute to improved quality of care and contraceptive choice.

### Theory of change

**Figure 1.**
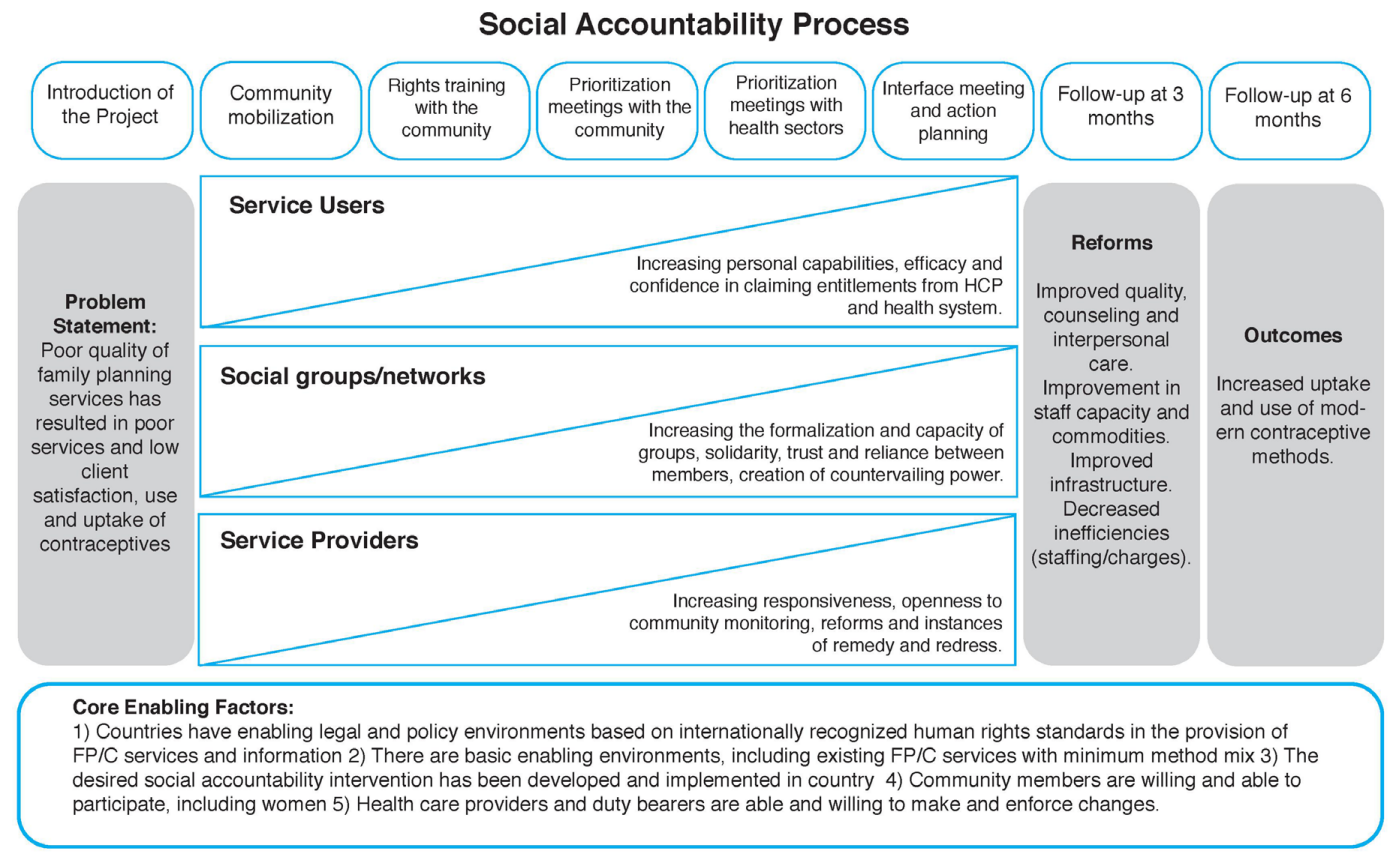
CaPSAI Project theory of change.

A theory of change (ToC) was developed for the CaPSAI Project ( Figure 1) based on studies on social accountability conducted by the Evidence Project ^13, 25^, the formative phase UPTAKE Project ^3, 27^ and the established literature in both FP/C programming and social accountability ^28– 32^. The ToC describes how a social accountability intervention is anticipated to address poor quality FP/C services that result in low client satisfaction and limited use and uptake of contraceptives. Engaging community members and health services actors in dialogues to discern challenges and developing action plans can lead to improvements in service quality, counseling, interpersonal care, staff capacity, and stock management. These, in turn, are expected to support full, free and informed choice and facilitate increased uptake and use of modern contraceptive methods.

The social accountability intervention is expected to create change by engaging group processes of health and civic education that raise awareness about health issues, service standards and local performance against them and then ways to engage to bring about change. At the individual level, the service user or potential user gains knowledge and is empowered to engage with the health system, both in terms of their own health seeking and their participation in community forums and in dialogues with authorities. Group engagement is interactive (e.g., prioritization, mapping) and supports social learning, creating a collective sense of shared (in)justice and can build confidence or assertiveness, which, in turn, can lead to more active collective engagement with providers, often through facilitated activities such as action planning and community monitoring of the agreed actions.

At the same time, these collective processes encourage health services actors to scrutinize themselves and alter their behavior in line with community concerns ^33^. Health systems actor response is critical for changes in the quality of service provision provided.

## Study protocol

The CaPSAI Project has been registered at Australian New Zealand Clinical Trials Registry ( ACTRN12619000378123, 11/03/2019) ^34^. The study was retrospectively registered following its classification as a clinical trial as per the WHO definition, which occurred during the continuing review of the protocol by the WHO ethics review committee a year after initial ethics approval. WHO International Clinical Trial Registry Platform (ICTRP) defines a clinical trial as any research study that prospectively assigns human participants or groups of humans to one or more health-related interventions to evaluate the effects on health outcomes.

### Study objectives

As complex interventions, social accountability interventions have several interacting components where it is difficult to identify ‘the active ingredients’ producing effects, and a range of outcomes is possible ^24^. The Medical Research Council’s (MRC) guidance on evaluating complex interventions recommends evaluating outcomes alongside process ^25^. To inform policy and practice, we need to know whether interventions ‘worked’ or not, but also how they were implemented, their causal mechanisms and how effects differed from one context to another.

The CaPSAI Project examines both the impact and processes of a social accountability intervention in the context of FP/C programming. Specifically, it aims to measure the effect of the intervention on contraceptive uptake and use. It also aims to understand the mechanisms and contextual factors that influence and generate these effects.

Two objectives were identified:

1. To develop more responsive quantitative measures for social accountability as well as show the relationship between social accountability and uptake of contraceptives and use and other FP/C behaviors;
2. To describe and examine how social accountability interventions are implemented and operationalized with a focus on understanding behaviors, decision-making processes, and the barriers and facilitators of change, with a view to generalizability.

To capture changes in contraceptive uptake and use, two designs are used:

i. *Contraceptive uptake*. A quasi-experimental pre-test post-test study, using a facility audit in both intervention and control facilities to determine the actual number of new users of contraception amongst women 15–49 in study catchment areas.
ii. *Contraceptive use*. A cohort of women who are new users of contraception, using standardized interview questions across both intervention and control facilities are tracked to measure changes in behaviors around contraceptive.

To measure the effects of the social accountability intervention, two additional designs were also used:

iii. *Social accountability outcomes*. An evaluation of the intermediary outcomes related to the social accountability intervention, using a questionnaire of psychometric scales among health care providers and service users in the intervention facilities only. We also assess the feasibility, acceptability, and validity of the instrument.
iv. *Process evaluation*. An evaluation of the implementation of the CaPSAI in intervention facilities, using predominantly qualitative methods and data sources to illuminate the processes contained in the social accountability intervention and enabling a fuller description of how the findings were produced.

### Study design and methods

1 The intervention

The study is implementing community and health provider driven social accountability interventions where service users and providers in each study country assess the quality of local FP/C services and jointly identify ways to improve the delivery and quality of such services. The CaPSAI intervention builds on community scorecard (CSC), citizen voice and action and citizen hearing methodologies that have been implemented and evaluated by other organizations ^13, 35, 36^. Eight common steps were distilled ( Table 2) and the essential principles of these steps were identified for adaptation to local contexts, emphasizing conceptual fidelity over standardization ^37, 38^. It is anticipated that this approach will allow for local adaptation while retaining fidelity across study sites and will result in generalizable findings.

**Table 2.** Eight standard steps of Community and Health Provider driven Social Accountability Intervention (CaPSAI).

Step	Description
**1. Introduction of the** **intervention to the** **community**	The implementation partner (a civil society organization) meets with local leaders, identifies stakeholders and sets up the infrastructure to deliver the social accountability intervention.
**2. Mobilization of participants** **for the intervention**	Community members, service providers, and other health services actors (duty bearers) are gathered by the implementing partner and introduced to the social accountability process.
**3. Health, rights and civic** **education with community** **participants**	The implementation partner shares information on health awareness and education, existing service standards and provides training on rights, good governance, and accountability. The group begins to rate existing services against rights-based standards and generate discussion about local priorities.
**4. Prioritization meeting with** **community**	The implementation partner distils themes and priorities raised by the community. The community groups then collectively score the issues and indicators and set priority areas for action.
**5. Prioritization meeting with** **duty bearers**	The implementation partner distils themes and priorities raised by the service providers. The providers then collectively score the issues and indicators and set priority areas for action.
**6. Interface meeting and joint** **action planning**	The implementation partner then holds a joint meeting between the community, the service providers and health services actors (duty bearers). Following the presentation of results from the prioritization meetings, the community groups and the service providers will aim to reach consensus on the ranking of the priority items and the actions required to address them. An action plan with assigned roles and responsibilities will be developed for the following 6- to 12-month period.
**7. First follow-up meeting** **with community and duty** **bearers at three months**	Priority areas and action items will be followed up with both the community and service providers. It is at this stage that change is anticipated on the part of health services actors and remedial actions have taken place which should be demonstrated in the monitoring activities. For any unresolved issues, these meetings present an opportunity to involve higher level duty bearers or third-party groups (media/politicians) to increase the pressure to act.
**8. Second follow-up meeting** **with community duty bearers** **at six months**	A second follow-up meeting will enable the monitoring of longer-range outcomes and on the remedy of unresolved issues raised in the first follow-up meeting. The community and service providers will continue to monitor the action plan for changes in relation to agreed priority areas.

2 Study setting

The intervention targets community members, health professionals and other duty bearers at both the community and facility level. Study outcomes are measured at the facility level.

Ghana and Tanzania were selected as the study countries based on the following selection criteria: existence of a national civil society organization (CSO) partner with local experience of delivering a social accountability intervention with the eight steps; low modern contraceptive prevalence rate; availability of contraceptive services at the point of contact by the client at no cost or where cost is not a barrier to access; an enabling environment in terms of the potential for the health system to respond to the social accountability activities; and the existence of formal structures linking the community with the health system (e.g. health committees). 

In each country, districts were pre-selected based on similar cultural, religious and socio-economic context, as well as presence of enough health facility offering FP/C services. Data on at least 20 facilities in the pre-selected districts, where there are no social accountability intervention in FP/C currently taking place, were gathered. From the initial list, eight intervention and eight control facilities were selected based on the following criteria: provision of contraceptive services; availability of the following methods: a barrier method, a short and long-acting method, emergency contraception, and at least referral for permanent methods in districts; no social accountability programs in FP/C currently underway. Criteria that include facility type and level, average number of service users and number of new users, will be used to match the study and control facilities.

3 Study design

The study design contains two parts: capturing changes in contraceptive uptake and use (i and ii) and evaluating the effects of the social accountability intervention (iii and iv). The overview of the study design is summarized in Table 3.

**Table 3.** Study overview table.

Data gathering activity	Facility audit	Cohort study	Cross sectional survey	Process evaluation
	Changes in contraceptive uptake and use		Effects of the social accountability process	
*Outcomes*	Contraceptive uptake (new users)	Contraceptive use (method discontinuation, continuation and switching)	Social accountability intermediate outcomes (service user and health provider empowerment; expansion of negotiated space)	Dose, reach and fidelity: Process evaluation and context mapping
**Participants**	Health facilities providing FP/C services	A cohort of new users	Health Care Providers, New and continuing users of facilities women accessing FP/C services in intervention facilities	Community and district participants and staff at key program/ implementation events N.B. The context mapping activity will be undertaken in both intervention and control sites.
**Study size**	Eight intervention and eight control facilities	Estimated 800 women over eight study facilities per arm	Health care professionals (two per site) and 750 women	Four process evaluations per country tracking between 8–12 intervention related events each
**Setting**	Intervention and control facilities	Intervention and control facilities	Intervention facilities	Intervention facilities N.B. The context mapping activity will be undertaken in both intervention and control sites (three interviews per district).
**Timing** *(N.B. Month 1–4* *was for preparing* *the study)*	Baseline: Month 5 Interim: Month 17 End-line: Month 29	Intake: Month 10–12 Check-up: Month 16–18 Follow up: Month 22–24	Pre-test: Month 5 Post-test: Month 17	8–12 data points tied to key events in the intervention facilities between Month 6 and Month 18. Eight events at four intervention facilities per country.
**Data sources**	Facility audit of FP/C facilities	Prospective cohort follow-up of new users	Cross sectional survey using a questionnaire of psychometric scales with women accessing FP/C and health care providers in intervention sites	Context mapping (three per district for both the intervention and control sites) In-depth interviews (three per observation) Observations (8–12 per process evaluation site) Document review

i. *Capturing changes in contraceptive uptake:* A quasi-experimental study utilizing an interrupted time series design with a control group (ITS-CG) is conducted to determine the actual number of new users of contraception amongst women 15–49 years old in eight intervention and eight control facilities per country. New users are defined as: never used an FP/C method (new acceptors); are switching from a traditional to a modern FP/C method (additional users); or are re-starting an FP/C method after a period of not using it for at least six months (additional users). The facility is the sampling unit.
  Contraceptive uptake in both the intervention and control arms is measured by comparing the level changes and trends over time during the study period. The numerator of the rate is measured using the facility audits provided by the intervention and control facilities, while the denominator of the rate, measured at the community level, is the number of women of reproductive age in the catchment area for the facility. The facility catchment area definitions used are those provided by Ghana Health Service in Ghana and the Ministry of Health in Tanzania. The catchment area study population size is collected from official statistics calculated on a yearly basis and, based on trend analysis, is expected to remain roughly constant throughout the study duration.
ii. *Capturing changes in contraceptive use*: A cohort of women, 15 to 49 years of age, who are new users of contraception and who are accessing FP/C services at the study facilities are tracked across both intervention and control facilities to measure changes in behaviors around contraceptive use such as method continuation, switching, contraceptive decision making, and client satisfaction. Method contraception discontinuation rate (first use) is estimated using a prospective cohort study design. The outcome of interest is time from starting modern contraceptive method use until when use is discontinued.
  Contraceptive method continuation (proportion of women in a cohort using the same contraceptive method after one year) and contraceptive switching (proportion of women in a cohort changing method within one year of initiation, out of those using any method at baseline/proportion of women, because they were not satisfied with the previous one) are measured.
iii. *Evaluating effects of the social accountability process:* To capture the intermediate outcomes outlined in the ToC (see Figure 1), such as empowerment of women and health providers and expansion of negotiated spaces, a cross-sectional survey using accountability-related psychometric scales is conducted at pre- and post-intervention phases. One survey is conducted with women accessing FP/C services in health facilities in the catchment areas where the social accountability intervention is being implemented. Another survey is conducted with health workers providing FP/C services in health facilities in the catchment areas where the social accountability intervention is being implemented.
iv. *Evaluating effects of the social accountability process:* A process evaluation of the implementation of the CaPSAI in intervention facilities uses a range of qualitative methods and data sources (non-participant observation [NON], in-depth interviews [IDI] and document review) and are conducted over the intervention and post-intervention period in four intervention facilities per country to trace the implementation, thus enabling a fuller description of how the outcomes were produced.
The process evaluation aims to respond to the following research questions: Was the intervention delivered as intended (per protocol) and did it reach the target audiences of health care providers, duty bearers, citizens and health service users? (Dose, reach and fidelity of the intervention.) What factors facilitate or hinder the implementation of the intervention in the study intervention facilities per country?
To understand the context where the intervention is conducted, a context mapping is administered to local leaders, health workers, and community representatives at three data collection points throughout the duration of the study in both the intervention and control districts. The context mapping aims to understand FP/C initiatives and other community participation programs to be considered alongside the findings. This will help to contextualize the implementation and effect of the study intervention.
To better understand how changes occurred in the course of the intervention, retrospective case studies exploring reported changes resulting from the intervention will be compiled.

4 Sample size

The sample size is calculated for each the four the study outcomes:

i. *Contraceptive uptake:* For sample size calculations, it is assumed that, at pre-intervention, the mean number of new users per facility per month would be similar in both the intervention and control arms. It is further assumed that in the control arm, the pre-test and post-test mean number of new users per facility per month, per arm will remain the same. Study sample size and power were estimated in two ways: with the facility as the unit of measurement for Poisson regression, and with the monthly data points as the unit of measurement for time series regression. With the facility as the unit of sampling and of analysis, and assuming two-time points of interest, at pre-test (or baseline) and at post-test, different sample size estimates were computed using a two-sided t-test with Type I error at 5% level, statistical power at 80%, and assuming equal variance. These various sample size estimates are provided assuming a constant denominator (included as an offset in the Poisson model) and a pooled variance for mean number of new users at pre-intervention in both arms of between 100 and 200 new users per facility, per month, and a difference in uptake per facility per month, at post-intervention of between 60 and 200 new users. Approximately, five to eight facilities are needed per intervention or control group to detect an approximately two-fold increase in the rate of new users with 80% power and allowing for a 5% Type I error. The monthly data points were used as the unit of analysis and, based on effect size and time periods, the simulation-based power calculation provided by Zhang *et al.*, 2011 ^39^ was used. Assuming an effect size of 2.0 (derived from 100 new users in control group and 200 new users in intervention group and a pooled standard deviation of 50) and an autocorrelation of up to 0.3, a total of 12 data points – the equivalent to six monthly pre-intervention data points and six monthly post-intervention data points – would be needed to achieve 80% power at p=0.05 statistical significance level ^39^.
ii. *Contraceptive use:* The discontinuation rate, being a time-to-event outcome and involving censoring, necessitates the use of sample size estimation methods for survival data. Different sample size estimates were computed for values of hazard ratio (intervention vs control) of 0.5, 0.6 and 0.7, and given values of the proportion of discontinuing use of modern contraception by end of the first year, in the control arm range from 30% to 60%. Sample size estimates were obtained using a Type I error at 5% level, accrual time of 0.01 years, and an exponential loss to follow-up of 20% that would enable a two-sided Logrank test to achieve 80% statistical power to detect the difference in discontinuation rates by the end of one year of follow-up. The final sample size was also adjusted for clustering ^40^ due to the intervention being offered at cluster level. Assuming an intra-class correlation of 0.05 and an average cluster size of 20 first users of modern contraception, resulting in a design effect of 2.0, the final sample size was doubled. The sample is estimated to be 800 women across five to eight study facilities per arm. The total sample is estimated at 1600 women distributed depending on the size of the facilities in the intervention and control arms in each country.
iii. *Social accountability intermediate outcomes:* In intervention facilities, health care professionals providing FP/C services (two per site at pre- and at post-intervention) and 750 women aged 15 to 49 years accessing FP/C services are interviewed pre- and post- intervention over an 18-month period. Sampling for the service users survey was calculated using *a priori* sample size calculation with the ratio of ten subjects per item ratio and guidance of more than 500 which equals a very good sample for validation ^11, 41^. The calculation is based on 75 items.
iv. *Process evaluation:* To answer the process evaluation research questions, key events related to the eight steps of the intervention, such as interface meetings, are observed by trained data collectors to capture data regarding the intervention process and surrounding contextual factors. IDIs are conducted with health providers, community members and other health services actors at the same key events. The anticipated minimum qualitative data capture overall is 174 IDIs (three per observation in four intervention facilities per country) and 64 NONs (one observation per activity, activities aligned to eight interventions steps in four intervention facilities per country).
  To better understand how changes occurred during the intervention, approximately 64 ‘case study of change’ interviews (eight per process evaluation across four intervention facilities per country) will be compiled at the end of the intervention. These will also incorporate IDIs, document reviews, and NONs. Interviews informing the case studies will be snowball-sampled from learning through other data collected throughout the process evaluation. Relevant documentation, such as implementation plans and reports, local action plans, budget commitments, local or national standards of service and onwards will be collected and included in the analysis.

5 Data collection

The data collection is described below for each of the study outcomes as above.

i. *Contraceptive uptake:* A standardized questionnaire is used to conduct the facility audits (see CaPSAI_FAU, *Extended data*) ^42^. The audit is conducted every 12 months during the study period to capture any changes at baseline, at mid-point (at 12 months) and at end-line (at 24 months). Monthly data on new users of contraceptives is recorded from six months prior to baseline and every month until end-line. Facility records are consulted to verify data on new users.
  The audit captures data on FP/C services available and their quality, as well as how well they are integrated into the other services provided by the facility. The audit also maps the community structures working with the facility or active within the facility catchment area. This instrument was developed using sections of the service availability and readiness assessment (SARA) tool specific to FP/C services (Sections 2–5) ^43^. The collected data are entered at each country using a customized web-based electronic data capture (EDC) system using the OpenClinica Enterprise platform for entering, cleaning and tracking the study data, developed by the WHO Department of Sexual and Reproductive Health and Research Statistics and Informatics Services (WHO/SRH/SIS) team in Geneva. The web-based data capture system is designed to ensure that only authorized staff can enter, change, or view data. The local study data management teams were trained in data collection, online data entry, and data management using the EDC system. The SRH/SIS team liaises with the local study team to coordinate and track case report form (CRF) completion and data queries.
ii. *Contraceptive use:* A cohort of women new to using contraception in both intervention and control facilities is followed-up over a 12-month period (starting at the end of the main intervention steps, i.e. end of step 6). Data is collected in real time using tablet-based standardized interview questions developed using OpenClinica Participate. The EDC system performs edit checks during the data capture process to notify immediately of potential errors and inconsistencies. The local study team keeps an updated log of screened and enrolled study participants.
  The two main instruments (intake and follow-up survey) collect data from new users of family planning services at the facility (see CaPSAI_ITS_CUI_FUI *Extended data*) ^42^. A mid-term check-up interview is used to reduce loss to follow-up and estimate continuation by confirming if the participants are still using a method and which method it is. The cohort study instruments are adapted from existing tools capturing demographic characteristics and contraceptive use ^44^, client satisfaction ^45^, contraceptive continuation ^46^. Exposure to or knowledge of the community and provider driven social accountability intervention is also captured.
iii. *Social accountability intermediate outcomes:* A cross-sectional survey using a questionnaire of accountability-related psychometric scales (see CaPSAI_XSU_XPV, *Extended data*) ^42^ is conducted with service users at the facilities receiving the intervention at pre- (before step 1 of the intervention) and post-intervention (starting after completion of step 8) and completed within three months. The study uses the CARE’s Women’s VOICES tool to measure these outcomes in sexual, reproductive and maternal health programs, adapted to assess FP/C programs ^47^. Some measures were adapted and new measures were added; these were field tested but did not undergo formative research. The service user survey is conducted using a tablet-based questionnaire to capture real-time data using OpenClinica Participate. Data are checked during data entry and potential data errors and inconsistencies are notified and resolved immediately. The health provider survey is conducted with paper-based CRF and collected data are entered at each country using a customized web-based EDC system using OpenClinica Enterprise platform. The screening and enrolment logs are kept at the sites by the local study team.
iv. *Process evaluation:* NONs and IDIs as part of the process evaluation are conducted during key events related to each of the eight intervention steps in four of the eight intervention facilities in each country.
  The NON instrument collects data on activities related to the CaPSAI and allow the capture of a broader picture of the intervention in action and the roles taken by citizens and health services actors in the social accountability process. The IDIs collect experiential data from key actors in the social accountability intervention including citizens, health care providers, and duty bearers (see ContextMapping_ProcessEvaluation, *Extended data*) ^42^. They are asked about their experience of the CaPSAI intervention, what they believe its impacts have been and their experience of intervention-related events. Document review is done as needed in all the eight intervention facilities.

6 Data analysis

The four main outcomes of interest will be analyzed in the following manner:

i. *Contraceptive uptake:* All potential confounders associated with all the facilities enrolled are recorded. An ITS segmented Poisson regression model will be used to estimate both the level changes in first-use modern contraception rate and changes in time trends for the rate of modern contraception first-use rates per 10,000 women-months, after the introduction of the intervention. The generalized estimating equation (GEE) Poisson segmented regression model will allow adjustment for correlation due to repeated observations from the same facilities, while also adjusting for important baseline family planning facility characteristics.
ii. *Contraceptive use:* For categorical variables frequencies and percentages will be reported, and for quantitative variables number of subjects, means, standard errors, medians, interquartile range, minima, and maxima will be reported. Rate of loss to follow-up for the one-year follow-up period will be computed by intervention arm. The one-year cumulative method discontinuation and method switching rates will be compared between the intervention and control arms. Because of the clustered nature of the outcomes, with intervention package designed at the cluster level, all time-to-event outcomes including loss to follow-up, method discontinuation, and method switching, will be analyzed with hazard ratios estimated from the marginal Cox model and/or shared Frailty models. The model will be adjusted for baseline potential confounders at participant and/or facility level. Two-sided tests, 5% significance levels and 95% confidence intervals will be used for all relevant parameters. Statistical Analysis System (SAS) Version 9.4 and R Version 3.3.3 software packages will be used for the statistical analyses. Open-ended questions will be listed and coded for meaningful comparisons of their distribution.
iii. *Social accountability intermediate outcomes:* Demographic data are collected and will be analyzed for representativeness. We will use difference-in-difference (DiD) and local average treatment effect (LATE) estimates to evaluate changes in social accountability intermediate outcomes at pre and post-intervention periods. The outcomes include knowledge and awareness of rights, decision making, self-efficacy, political capabilities and collective efficacy among women and health providers. For the validity of the service users scale, an analysis of the distribution of responses for individual items will be conducted and those with no variability will be removed. A reliability analysis will follow on each proposed scale, with items removed in accordance with standard procedure. The distribution of overall and dimension scores will be analyzed by calculating mean scores and standard deviations (SD). Floor and ceiling effect will be assessed. To determine internal consistency, Cronbach’s Alpha analysis will be conducted with the use of standard thresholds of 0.60 for acceptable reliability and 0.70 to be good or high reliability. Re-test will be assessed using intra-class correlation coefficient. We will be assessing the feasibility and acceptability of the instrument by assessing the time taken to complete the questionnaire and ease of use through analysis of the completion rate and range of missing answers.
iv. *Process evaluation:* Process evaluation data will be synthesized to produce an overall summary of the outcomes of the process evaluation, and this will be supplemented with other outcomes data. NONs will be transcribed. IDIs are audio-recorded and transcribed/translated into English, where applicable. A common data analysis plan will be used that describes the synthesized analysis of the data. It enables evaluation at a country level and comparability across countries. This is necessary to be able to answer questions about the intervention and its effects. All qualitative components will be analyzed in a single qualitative data analysis database using NVivo, a qualitative analysis software. Data will be thematically coded at country level, although a single yet iterative code list will be used to enable cross country comparisons where applicable. Throughout analysis of the process evaluation data, research questions may be identified to be explored in the process evaluation. The case studies of change can be used to consolidate and synthesize data, providing information on case studies of interest (e.g., high performing in comparison to low performing sites or facilities). Document review, which comprises collecting materials or forms of documentation, might be useful to help develop the case studies of change.

7 Ethics

CaPSAI Project master and country protocols (Project ID A65896) were approved by technical and ethics review committees at the World Health Organization (WHO). Additionally, the country protocols were reviewed and approved by the Population Council Institutional Review Board (exemption approval - # EX201714) and Ghana Health Service Ethics Review Committee (GHS-ERC:009/08/2017) in Ghana. In Tanzania, the protocol has been approved by Ifakara Health Institute Institutional Review Board (IHI/IRB/No:18-2018 and IHI/IRB/AMM/No:03-2019) and the National Institute of Medical Research (NIMR) review board (NIMR/HQ/R.8a/Vol.IX/2668), as well as the NIMR/Mbeya Medical Research and Ethics Review Committee (GB.152/377/01/214a).

The study adheres to the highest ethical standards, and to the current international and local legislation pertaining to research governance. All study participants undergo informed consent procedures. For low literacy respondents expressing interest in participating in the study, the data collector requests the respondent to identify a trusted literate witness to be part of the informed consent process or to come back to the facility with the trusted literate witness to go through the consent. For adolescents, the consenting/assenting and interviewing may a be a two-step process. The researcher explains the study and informs the adolescent that their parent or guardian needs to be informed about the study and give their consent unless they are emancipated adolescents. If the adolescents agree to participate and they are accompanied by their parents or guardian, consent is obtained from the parents/guardian, followed by assent from the adolescent. If the parent/guardian is not present at the facility, they are invited to come back to the facility with their parent/guardian to go through the consent and assent process separately. Emancipated adolescents are able to give informed consent in both countries according to local regulatory bodies. Written informed consent is not taken for the non-participant observation but a group consenting process is followed. Participants are provided with a copy of a study information sheet (see CaPSAI Informed Consent Forms- *Extended data*) ^42^, informed that the activity is part of a research study and are offered the opportunity to leave (to opt-out of being observed) or to ask questions about the study aspects. 

All identifying information is removed from transcripts and stored separately with access restricted to the research team. All transcripts are stored electronically in password-protected online services, and physical documents are securely stored at the principal investigators’ institutions.

8 Dissemination

The study results will be disseminated in participating districts and at the national level as well as with the global FP/C communities through publications and presentations to contribute in addressing the gap in evidence on integration and measurement of social accountability interventions for FP/C. An implementation manual providing guidance on social accountability for FP/C will be developed based on the findings of the study.

De-identified country-specific data will be available one year after primary manuscripts have been published as agreed on a Data Transfer Agreement. De-identified country-specific data will be available for 10 years after that. As this is a multi-country study, the ownership of country-specific data will be at country-level. Request for de-identified data may be submitted to Primary Sponsor (WHO SRH/HRP Research) or Principal Investigators, and data will be shared contingent on approval by the internal review and approval by local internal ethics review board.

9 The CaPSAI Project team

The study and intervention design were jointly conceptualized and designed by the Department of Sexual and Reproductive Health and Research, WHO, which includes the United Nation Development Program/United Nations Population Fund/United Nations Children’s Fund/WHO/World Bank Special Programme of Research, Development and Research Training Human Reproduction (WHO SRH/HRP) and the Evidence Project. Overall oversight for the CaPSAI is the responsibility of WHO SRH/HRP. WHO SRH/HRP is leading the research component.

Population Council in Ghana and Ifakara Health Institute (IHI) in Tanzania were selected to conduct the research study based on the strength of experience and expertise of the Principal Investigator and the existing infrastructure, capabilities and stellar track record. Ghana Integrity Initiative (GII), which is the Local Chapter of Transparency International (TI) in Ghana, and Sikika in Tanzania were the national CSOs selected to implement the intervention based on their previous experience of conducting social accountability interventions in health.

### Study status

Baseline data collection was initiated in March 2018 and endline data collection will be conducted in March to April 2020.

## Discussion

### Measuring impact of a process – ITS analysis

Contraceptive uptake is an outcome of interest and will be evaluated using ITS analysis. ITS study design is increasingly being used to evaluate public health interventions that are introduced over a defined time period and target population-level health outcomes ^48^. Randomized control trials (RCTs) are considered as the gold standard in studying causality as well as being efficient in terms of minimizing the risk of bias, but they are not always feasible due to ethical or practical reasons. ITS-CG design is considered the next-best design after RCT.

### Researching complexity

Social accountability as a complex process poses challenges in neatly linking intervention to the outcomes. The MRC guidance on evaluating complex interventions calls for a combined evaluation of outcomes alongside process ^25^. It is necessarily to know whether the intervention ‘worked’ but also how they were implemented, their causal mechanisms and how effects differed from one context to another. The MRC recommends that such evaluations: identify how the intervention works in everyday practice; explain the discrepancies between expected and observed outcomes; understand a wide range of effects; and determine how the intervention effects vary among recipients, in different sites, over time ^25^. The CaPSAI Project study design takes into account these noted complexities ( Table 4).

**Table 4.** Accounting for complexity in the Community and Provider driven Social Accountability Intervention (CaPSAI) study design.

Dimension of complexity (MRC)	CaPSAI dimension	MRC recommended design features	CaPSAI study design features
A large number of interactions between components within the intervention ^24^.	CaPSAI intervention requires separate and joint activities with community organizations, health providers, and duty bearers to produce an effective space for collective action and change.	A theoretical understanding of how the intervention causes change ^24^.	CaPSAI developed a theory of change ( Figure 1)
A number of behaviors required by those delivering or receiving the intervention ^24^.	Behavior change in varying degrees on the part of community members, health providers, and duty bearers is required for effects to take hold.	A process evaluation design to study the implementation process to address ‘how’ the intervention worked in practice and better understand the ‘active ingredients’ ^24, 25^.	Process evaluation is a main component of the study design
A number of groups or organizational levels targeted by the intervention ^24^.	CaPSAI intervention targets community members, health care providers and duty-bearers at the community and facility level.	A larger sample size and cluster rather than individual level designs ^24^.	Evaluation and sampling at both the community (individual) and health facility level (cluster).
Numerous and variable outcomes ^24^.	The primary outcomes include an increase in contraceptive uptake and indicators of contraceptive use alongside intermediary outcomes such as increases in social capital, collective efficacy, and empowerment.	Use of a range and mix of measures and methods to capture complexity and unintended consequences ^24, 25^.	A range of methods and instruments aim to capture the primary and intermediary outcomes as well as the implementation process itself.
A degree of flexibility or tailoring of the intervention is permitted ^24^.	Implementation should maintain conceptual fidelity to the eight standard steps but allows for local adaptation to produce the effects.	Fidelity should be considered ‘functionally rather than compositionally ^32^ to allow interventions to be responsive to context while still being meaningfully evaluated ^24, 25, 37^.	The process evaluation and combination of research instruments has been designed to capture functional fidelity in the implementation

MRC, Medical Research Council.

### Ethics issues

The intervention can be described as being an uncontained and unpredictable social process and some risks may arise, such as the occurrence of social harms from participating in the intervention activities as well as identification of “unusual occurrences” or “patterns of problems” during meetings. The implementation team will aim to minimize possible incidents of social harm by doing a thorough introduction of the project at various levels of administration (regional, district, ward or area).

Although the study is expected to be of minimal risk as no biological samples are collected, no drugs are provided, and no medical procedures are conducted, patients and health workers being interviewed may find some questions sensitive or embarrassing. While narrating their life stories they may become emotional as they remember important events. Interviewers will possess basic counseling skills to deal with such risks and will be able to refer HIV-positive patients to counselors within the local HIV care programme if required. There are no direct benefits to the respondents from participating in this study.

### Study limitations

There are limitations to the study design.

There is a risk of under-estimation bias if, despite intervention coverage in the study facility/catchment area, some women choose to go to one facility over another facility in the (same catchment area).

The cohort study may experience limitations due to loss to follow up, the large numbers needed for the sample and willingness to participate. One of the eligibility criteria will be willingness/anticipation of not moving outside the study area/district during the period of study. The check-up interviews at six months will also help address loss to follow-up and recall bias.

We anticipate that the research findings and conclusions from this study can be taken from the sample population to the population at large. The study is designed to capture the statistical probability from the quantitative studies/reliability of findings.

The measures used for the social accountability cross-sectional surveys have been adapted from related work; adaptations made to these items and response options may change the psychometric properties of the original measures ^47^. Attributing changes to the intervention directly poses a challenge. Respondent bias may pose a challenge as some measures discuss sensitive material.

Due to the lack of feasibility and budget to conduct intensive process evaluation data collection across all eight intervention sites, it was decided to do an in-depth focus on four implementation sites where NONs and IDIs will be conducted during key intervention activities. This allows for a very thorough understanding of implementation in four sites but compromises an overall view of the implementation by not collecting as much data in the other four intervention sites. However, documentation regarding intervention activities in all intervention sites, such as plans and reports created by the implementing CSOs, are analyzed to evaluate the dose, reach and fidelity of the implementation of the intervention across all sites.

## Conclusions

The completion of the study aims to provide researchers and implementers with the means to better measure and understand the mechanisms and contextual factors that influence social accountability processes in reproductive health, adding important findings to the evidence base. The study is expected to systematically assess social accountability processes, provide evidence of their effectiveness in improving FP/C services and demonstrate a relationship between the social accountability intervention and changes in contraceptive uptake and use. Though the findings may be context-specific, we anticipate this study will make a valuable and helpful contribution to the field with application to the development of FP/C programming in settings with significant unmet need.

An implementation manual for integrating social accountability processes in family planning service provision based on a set of effective practices will be developed and disseminated. At the end of the project, psychometric scales measuring social accountability intermediate outcomes in FP/C will be validated, ensuring more responsive quantitative measures for social accountability. The implementation and evaluation of the CaPSAI intervention are also expected to contribute to the existing evidence of conducting evaluations of complex interventions and inform reporting standards that enable a clear description of the interactions between research and implementation components and how these influence study results.

## Data availability

### Underlying data

No underlying data are associated with this article.

### Extended data

Figshare: CaPSAI Project - Extended Data. https://doi.org/10.6084/m9.figshare.11743206 ^42^

This project contains the following extended data:

- CaPSAI Informed Consent Forms-GH.pdf (sample consent forms and participant information sheets)
- CaPSAI_FAU.pdf (facility audit questionnaire)
- CaPSAI_ITS_CUI_FUI.pdf (intake, mid-term check-up and follow-up interview study instruments)
- CaPSAI_XSU_XPV.pdf (social accountability questionnaire)
- ContextMapping_ProcessExaluation.pdf (process evaluation NON and IDI instruments)

Data are available under the terms of the Creative Commons Attribution 4.0 International license (CC-BY 4.0).
