# Supplementary material for: Rationale and design of a complex intervention measuring the impact and processes of social accountability applied to contraceptive programming: CaPSAI Project
Source: Gates Open Res. 2020 Dec 10;4:26. Originally published 2020 Mar 5. [Version 2] doi: 10.12688/gatesopenres.13075.2 (PMC7541905; doi:10.12688/gatesopenres.13075.2)
Supplement: Peer review history

# Peer review: Reviewer response for version 1

**DOI:** 10.21956/gatesopenres.14213.r29403

**Published:** 2020-10-07

**Authors:** Anita Makins

**Affiliations:** 1 Department of Women's and Reproductive Health, University of Oxford, International Federation of Gynecology and Obstetrics, Oxford, UK

This report details the protocol designed for the CaPSAI Project. The CaPSAI project is a well thought out complex study looking at whether social accountability methods can positively influence the provision and uptake of FP/C services. The theory of change is appropriate and the research methodology is adequate to assess impact in that it is multifaceted, using both quantitative and qualitative methodologies. On the whole the authors are to be congratulated on their study protocol and I look forward to the outcomes in the next few years.

 I have little to add other than some specific comments regarding details in the manuscript which I have outlined below.

 1. The intervention:

- There is little explanation on the actual intervention. Table 2 gives an overall view of eight standard steps but it would be good to outline better the specifics of the assessments which the community groups will do regarding their local FP/C services. Given there are different partners involved in the implementation its important these processes are standardised. Please expand on this.

2. Study setting:

- There is no comment regarding whether the 8 sites are in rural or urban areas? Also what kind of health facilities, district general hospitals, clinics or referral centres, patient flow in these centres etc.? I realise these are questions which are asked in the audit tool but I just wonder whether if the units are widely different, this will effect outcomes and needs to be taken into account when comparing the sites. Given the intention to make comparisons with the control sites have these been chosen specifically to be comparable in terms of the characteristics I mention? Perhaps there needs to be more of an explanation on how the 8 facilities in each arm were chosen and whether they are comparable to the control sites?

3. Study design:

- Table 3: Instead of using numbers 1.1, 1.2 etc., for your row on data I would write out the specific outcomes so people don’t have to keep referring back otherwise it is not clear what you are measuring. Also you are not consistent in the text with using the same numbers (sometimes you use (i), (ii) and sometimes you use 1.1, 1.2.). Its easier if you consistently use the same numbers for the outcomes measures.
- Study size – 1.2 : 800 women over 8 study facilities per arm – this is not clear. In 2.1 : why then 750 women when looking at effects of the social accountability process?
- Timing – 1.2 : what do you mean by intake? And 2.2 also not clear.

4. Sample Size:

- ‘The sample is estimated to be 800 women across five to eight study facilities per arm.’ Please make it clear how many sites you will have and how many women were recruited in each site. By ‘per arm’ do you mean control and intervention arms – if so, say this.

Is the study design appropriate for the research question?

Yes

Is the rationale for, and objectives of, the study clearly described?

Yes

Are sufficient details of the methods provided to allow replication by others?

Yes

Are the datasets clearly presented in a useable and accessible format?

Not applicable

Reviewer Expertise:

My expertise is in global women's health and I am a specialist Obstetrician & Gynaecologist.

I confirm that I have read this submission and believe that I have an appropriate level of expertise to confirm that it is of an acceptable scientific standard.

# Response

**Published:** 2020-11-25

**Authors:** Joanna Cordero

**Affiliations:** World Health Organization, Switzerland

**Comment 1:** There is little explanation on the actual intervention. Table 2 gives an overall view of eight standard steps but it would be good to outline better the specifics of the assessments which the community groups will do regarding their local FP/C services. Given there are different partners involved in the implementation its important these processes are standardised. Please expand on this.          

**Response:** In each setting, the partners implementing the intervention undertaken activities that will achieve the intented outcome of each step. For example inclusive community mobilization may involve large community meeetings in one context whereas it might involve more targetted engagements with specific groups in other settings. How the steps are implemented in certain settings should be different as it responds to local conditions. The focus on conceptual fidelity over standardization of interventions is in line with complex intervention (Moore et al., 2015; Craig et al. 2013).

**Comment 2:** There is no comment regarding whether the 8 sites are in rural or urban areas? Also what kind of health facilities, district general hospitals, clinics or referral centres, patient flow in these centres etc.? I realise these are questions which are asked in the audit tool but I just wonder whether if the units are widely different, this will effect outcomes and needs to be taken into account when comparing the sites. Given the intention to make comparisons with the control sites have these been chosen specifically to be comparable in terms of the characteristics I mention? Perhaps there needs to be more of an explanation on how the 8 facilities in each arm were chosen and whether they are comparable to the control sites?      

**Response:** Additional information has been included in the site selection description including matching criteria.

**Comment 3: **Table 3: Instead of using numbers 1.1, 1.2 etc., for your row on data I would write out the specific outcomes so people don’t have to keep referring back otherwise it is not clear what you are measuring. Also you are not consistent in the text with using the same numbers (sometimes you use (i), (ii) and sometimes you use 1.1, 1.2.). Its easier if you consistently use the same numbers for the outcomes measures.

**Response: **The data gathering activity is now included on top of table 3.

**Comment 4:** Study size – 1.2 : 800 women over 8 study facilities per arm – this is not clear. In 2.1 : why then 750 women when looking at effects of the social accountability process?       

**Response: **Sample size calculations are specific to the main outcomes. For  contraceptive use, the sample size calculation is based on estimates of discontinuation rates described in the text. For the effects of social accountability process (Social accountability intermediate outcomes) the sample size calculation is described in the text. Sampling for the service users survey was calculated using a priori sample size calculation with the ratio of ten subjects per item ratio and guidance to validate the study instrument.

**Comment 5: **Timing – 1.2 : what do you mean by intake? And 2.2 also not clear. 

**Response:** Intake interview for the cohort study refers to the first data collection point, which is after the completion of step 6 of the intervention.

**Comment 6:** The sample is estimated to be 800 women across five to eight study facilities per arm.’ Please make it clear how many sites you will have and how many women were recruited in each site. By ‘per arm’ do you mean control and intervention arms – if so, say this.

**Response: **The number of women per facility depends on the size of the facility. It is clarified that "The total sample is estimated at 1600 women across the intervention and control arms in each country".

# Peer review: Reviewer response for version 1

**DOI:** 10.21956/gatesopenres.14213.r28651

**Published:** 2020-04-02

**Authors:** Angela Bailey

**Affiliations:** 1 American University, Washington, DC, USA

This study design paper is similar to a ‘pre analysis plan’ in social science RCTs which codify predetermined analytical parameters of a research study – outcomes, definitions, measurement tools, and analysis plans. It serves this purpose.

 Overall strengths – Clear mixed methods approach to data collection and proposed analysis of findings. Study designed to assess and enhance understandings of both 'processes' and 'outcomes' which is also valuable. Paper offers definitions of indicators, overview of the intervention studied, and links to extended survey tools. This transparency in survey tools and ultimately calculations will enhance the utility and applicability of the research findings to broader audiences across disciplines of public health and political science. Very much appreciate the distinction between ‘standardization’ and ‘conceptual fidelity’ and the study’s prioritization of 'conceptual fidelity' -- this makes a great deal of sense when trying to facilitate dynamic processes such as the "intervention" studied. It will be very important to bring this out in any eventual analysis and to apply this to whatever tools or guidance notes are produced (as discussed in the dissemination section).

 Possible challenges – The ‘tradeoffs’ between interventions that are measurable versus interventions that have all the component parts to create change are fundamental challenges in studies of health accountability (to use a broad term). The section on ‘study limitations’ focuses on the limits of the research methods, but does not reflect possible limits of this intervention itself. Both the intervention and the study risk perpetuating the ‘local level’ interface of users/potential users and service providers as necessary and sufficient to achieve outcomes (both in terms of FP/C uptake and continued use and the empowerment/accountability dimensions measured using psychometric scales). The extended data survey tools are quite long, the authors could make it easier to quickly identify or link the specific 'extended data' file to the parameter under discussion. For example, I was looking through the tools for more on the ‘psychometric scales' and it was not straightforward to identify that among the multiple files. The distinction between “process” and “intervention” could be made more consistently throughout the text (or in any papers on the findings). In the text, if possible link descriptions of measures/numerators/denominators to specific source files within the extended data documents for easier tracing because the devil is truly in the details.

Is the study design appropriate for the research question?

Yes

Is the rationale for, and objectives of, the study clearly described?

Partly

Are sufficient details of the methods provided to allow replication by others?

Yes

Are the datasets clearly presented in a useable and accessible format?

Not applicable

Reviewer Expertise:

Governance, accountability, advocacy, health systems

I confirm that I have read this submission and believe that I have an appropriate level of expertise to confirm that it is of an acceptable scientific standard.

# Response

**Published:** 2020-11-25

**Authors:** Joanna Cordero

**Affiliations:** World Health Organization, Switzerland

**Comment 1: **Possible challenges – The ‘tradeoffs’ between interventions that are measurable versus interventions that have all the component parts to create change are fundamental challenges in studies of health accountability (to use a broad term). The section on ‘study limitations’ focuses on the limits of the research methods, but does not reflect possible limits of this intervention itself. Both the intervention and the study risk perpetuating the ‘local level’ interface of users/potential users and service providers as necessary and sufficient to achieve outcomes (both in terms of FP/C uptake and continued use and the empowerment/accountability dimensions measured using psychometric scales).    

**Response: **We have used a conceptual fidelity approach in the intervention design to avoid this 'trade-off' between a measurable intervention and an intervention that can bring about change in a local context. We have another paper dedicated to the issue of conceptual fidelity in developing the intervention that is about to be published.

**Comment 2: **The extended data survey tools are quite long, the authors could make it easier to quickly identify or link the specific 'extended data' file to the parameter under discussion. For example, I was looking through the tools for more on the ‘psychometric scales' and it was not straightforward to identify that among the multiple files.            

**Response:** We have included specific file names that are uploaded as extended data in the text.

**Comment 3: **The distinction between “process” and “intervention” could be made more consistently throughout the text (or in any papers on the findings).   

**Response:** We reviewed the text and have addressed the issue. Social accountability process is used to refer to the process itself. Program is used for implementation of social accountability either in study settings or not elsewhere. Social accountability intervention now only refers to the implementation of social accountability as a study intervention, including in the context of CaPSAI.

**Comment 4:** In the text, if possible link descriptions of measures/numerators/denominators to specific source files within the extended data documents for easier tracing because the devil is truly in the details.

**Response: **We have included specific file names that are uploaded as extended data in table 3 to specify sources for each main outcome.
